# Supplementary material for: Maturation of Aluminium Adsorbed Antigens Contributes to the Creation of Homogeneous Vaccine Formulations
Source: Vaccines (Basel). 2023 Jan 11;11(1):155. doi: 10.3390/vaccines11010155 (PMC9862877; doi:10.3390/vaccines11010155)
Supplement: Supplementary file 1 [file vaccines-11-00155-s001.zip › vaccines-2134072-supplymentary.pdf]

vaccines

MDPI

Supplementary materials of:

Maturation of Aluminium Adsorbed Antigens Contributes to the Creation of Homogeneous Vaccine Formulations

Donatello Laera [1,*,†], Camilla Scarpellini [1,2,†], Simona Tavarini [3], Barbara Baudner [1], Agnese Marcelli [1], Carlo Pergola [1], Malte Meppen [1] and Derek T. O'Hagan [4]

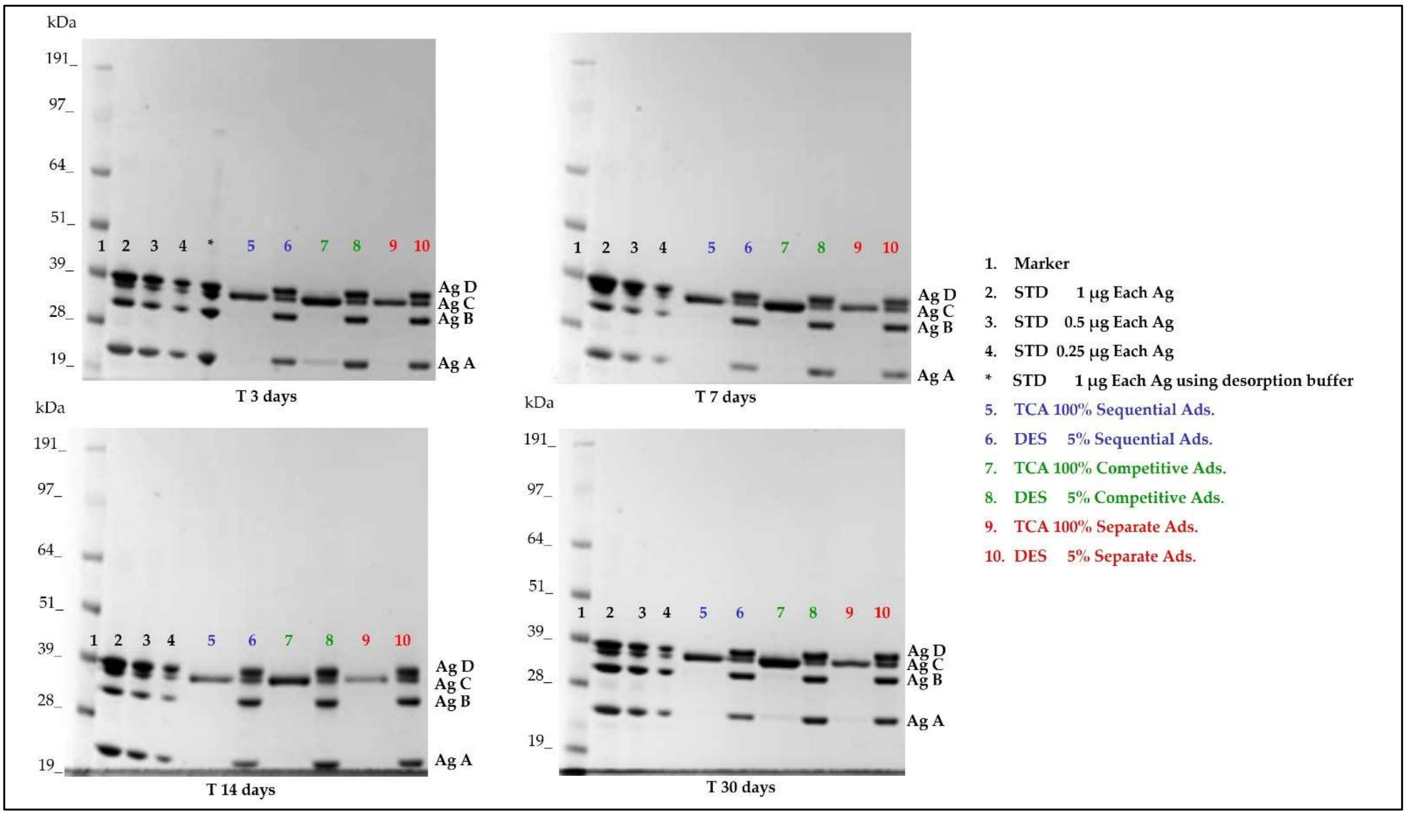

Figure S1. SDS-PAGE results respectively at time points 3, 7, 14 and 30 days which show antigens identity in respect to standard controls and degree of antigens adsorption after steps of centrifugation and separation of supernatants from AlumOH pellet.. STD: Standard antigens solution of known amount; Ag: Antigen; TCA: Supernatant precipitated with Tri-Chloro Acetic Acid after steps of centrifugation and AlumOH separation; Ads: Adsorption; DES: AlumOH desorbed with desorption buffer following steps of centrifugation and supernatant separation.

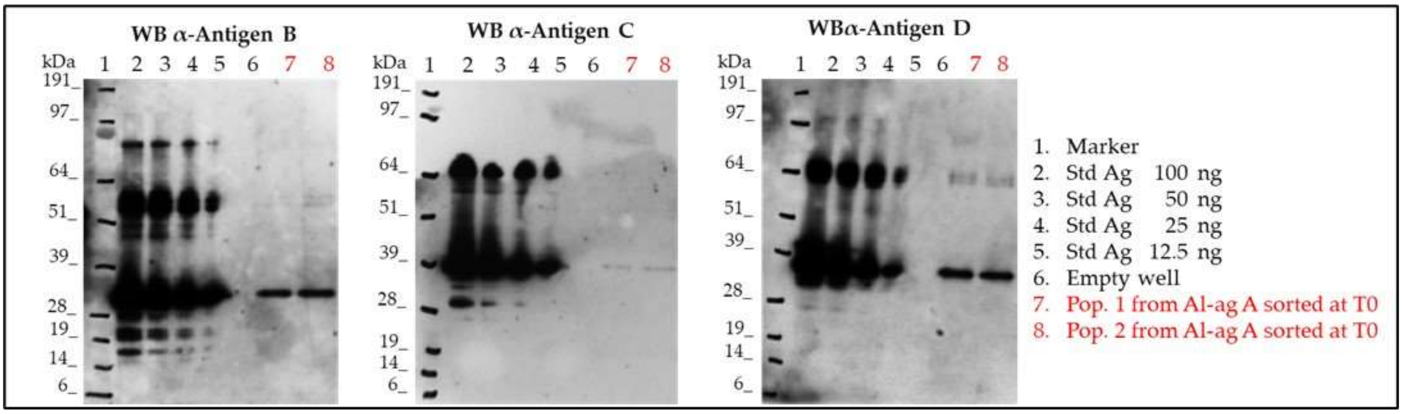

Figure S2. Western blot results respectively for antigen B, C and D of sorted AlumOH populations P1 and P2 after Flow Cytometry staining, for orthogonal evaluation of delivered antigen content. STD: Standard antigens solution of known amount, P1 and P2: AlumOH population sorted from Separate ads. sample at T0 after antigen A staining (see Figure 7).

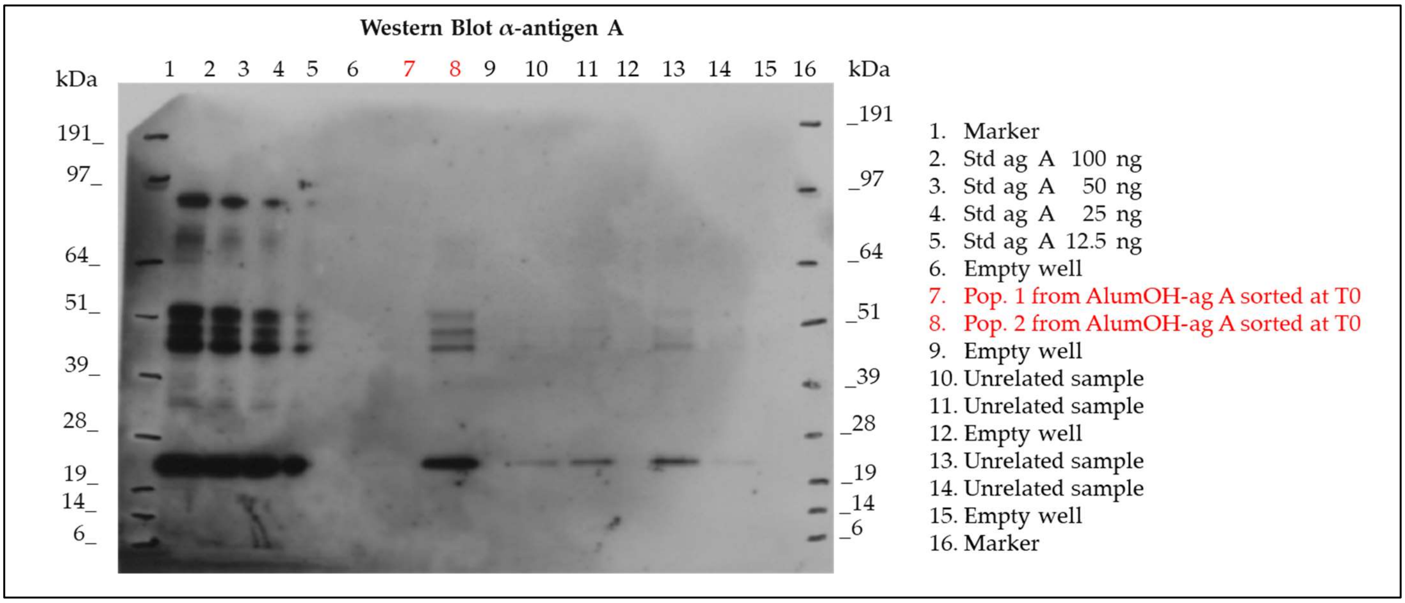

Figure S3. Whole western blot of figure 9.
